# Calibration-Free Analysis with Chronoamperometry at Microelectrodes

**DOI:** 10.1021/acs.analchem.4c01645

**Published:** 2024-09-03

**Authors:** Valdomiro S. Conceição, Douglas P. M. Saraiva, Guy Denuault, Mauro Bertotti

**Affiliations:** ‡ Department of Fundamental Chemistry, Institute of Chemistry, University of São Paulo-USP, São Paulo, 05508-000, Brazil; # School of Chemistry, University of Southampton, Highfield, Southampton SO17 1BJ, U.K.

## Abstract

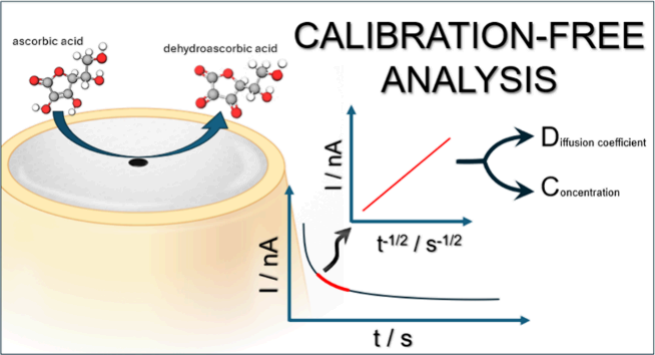

Analytical methods are crucial for monitoring and assessing the concentration of important chemicals, and there is now a growing demand for methodologies that allow miniaturization, require lower sample volumes, and enable real-time analysis in the field. Most electroanalytical techniques depend on calibrations or standards, and this has several limitations, ranging from matrix interference, to stability problems, time required, cost and waste. Therefore, strategies that do not require standards or calibration curves greatly interest the analytical chemistry community. Here, we propose a new quantification method that does not rely on calibration and is only based on a single chronoamperometric curve recorded with a microelectrode. We show that satisfactory analytical information is obtained with just one chronoamperometric experiment that only takes a few seconds. We propose different data treatments to determine the unknown concentration, we consider the experimental conditions and instrument parameters, we report how parallel reactions affect the results, and we recommend procedures to implement the method in autonomous sensors. We also show that the concentration of several species can be derived if their *E*° values are sufficiently far apart or the sum of all concentrations if the *E*° values are too close. The proposed method was validated with a model redox system then further evaluated by determining ascorbic acid concentrations in standard solutions and food supplements, and paracetamol in a pain killer. The results for ascorbic acid were compared with those obtained by coulometry, and a good agreement was found, with a maximum deviation ca. 10.8%. The approach was also successfully applied to ascorbic acid quantification in solutions with different viscosity using ethylene glycol as a thickener.

## Introduction

Electroanalytical methods are crucial for monitoring and assessing the concentration of chemical species. Such methods are routinely used to detect and quantify contaminants in the air,^1^ water,^2−7^ and soil,^8^ to monitor biological markers in complex samples,^9^ to detect and collect evidence for criminal investigations,^10^ to ensure the efficacy and safety of pharmaceuticals,^11−13^ and to monitor the quality of foods,^14,15^ and beverages.^16^

Most common electroanalytical techniques depend on calibrations or standards; this increases costs and hinders the development of point-of-care and continuous applications. Although widely used, electrochemical quantification using external calibration can encounter challenges when dealing with complex samples containing interfering or poisoning species. Point-of-care glucose sensors that rely on external calibration nicely illustrate the increased complexity and costs associated with this approach. In this case, each batch of glucose sensing strips is calibrated in the factory and a calibration chip included in the batch must be connected to the sensor electronics by the consumer. For continuous glucose monitoring devices, the calibration problem is even worse with the consumer having to calibrate the equipment every few days.^17^

Other approaches, such as standard addition where calibration curves are produced by adding known amounts of an analyte standard to the sample,^18^ can overcome some of the limitations inherent to external calibration, mainly with regards to matrix interference. This strategy is useful to derive the concentration of an analyte in a highly complex sample. However, it is lengthy, and it uses significant sample volumes, which can be a limiting factor in cases involving forensic or biological samples.^18^ Furthermore, the standard addition method is not applicable to continuous monitoring and in-line quantification protocols since samples must be drawn from the process line and discarded after analysis.^19^

In general, calibration-based methods involve reagents consumption, waste generation, errors from matrix effects, and extra time to produce analytical plots. Analytical methods that do not require calibration or standards overcome most of these issues. To our knowledge, few calibration-free electroanalytical techniques have been reported to date.

In coulometry, one of the classical calibration-free electrochemical methods, the amount of target analyte in the sample is derived from the total charge needed for a complete redox conversion. As an absolute method, coulometry does not require calibration or standards and can achieve high accuracy if the current efficiency is 100%. Calibrations are only required in specific cases, such as coulometric titrations involving the analysis of the water content with Karl Fischer reagent. However, the technique is highly prone to interferences, and it is necessary to guarantee that only the analyte undergoes the reaction of interest. Moreover, being an exhaustive method, the sample is consumed and thus not available for subsequent investigations. Finally, coulometry is not suitable for continuous or fast analysis as measurements can take tens of minutes. Thus, coulometry can be used for niche analysis with high accuracy but is often not recommended for many analytes and matrices.

Most of the calibration-free electroanalytical techniques reported in the literature rely on the unique properties of microelectrodes, such as the radial mass transport and its derived steady-state current. In one example, Daniele et al. used Hg^2+^ as an internal standard in the anodic stripping voltammetric detection of Pb^2+^ and Cu^2+^^19^ and proposed an equation correlating the analyte concentration with known quantities, such as the number of electrons involved in the reactions, the measured oxidation charges, and the diffusion coefficient of both target analytes which were determined in a parallel experiment or found in the literature.^19^ In a subsequent study, the same group employed the stripping procedure at microelectrodes to determine sulfide ions in solution; however, the independent determination of the diffusion coefficient was still required.^20^ In a parallel study, they exploited the properties of microelectrodes to determine Cd^2+^, Pb^2+^ and Cu^2+^ concentrations in rain samples without calibration.^21^ Whereas these approaches relied on a single microelectrode, Giraud and co-workers proposed a different calibration-free protocol to determine silicates in seawater samples. Their experimental approach involved two Au disc electrodes, a microelectrode and a macroelectrode, and two successive chronoamperometric measurements from which the diffusion coefficient and the target analyte concentration could be determined through appropriate equations.^22^ Although more cumbersome, their method is still simple and could be easily implemented on a sensor platform for continuous monitoring.

In this work we report a calibration-free electrochemical method, which is accurate, fast, low-cost, and only requires one experiment with one microelectrode. In the following, we present the theoretical background which underpins the approach, the methodology to select the optimum experimental time scale, the parameters and conditions used to perform the experiments, and the results. In the latter, we validate the method by determining the concentration of hexaamineruthenium, ascorbic acid, and paracetamol. To demonstrate the ability of the technique to operate with samples of unknown viscosity, we report the determination of ascorbic acid concentrations in the presence of a thickener. We finish the article with a thorough discussion of the instrumental and operational conditions necessary to ensure the accuracy of the results.

## DEVELOPMENT OF THE CALIBRATION-FREE METHOD

In the following we develop the theoretical framework of the calibration-free methodology for disc-shape microelectrodes, microdiscs, since these are the most common microelectrodes used in electroanalytical chemistry. For this, we make three assumptions: 1) that the sample solution contains a species of interest, R, that undergoes a fast oxidation at the electrode surface, 2) that the solution is quiescent, and 3) that it contains a large concentration of inert electrolyte. Together, these assumptions ensure that the electrochemical oxidation of R is purely diffusion controlled since neither electron transfer kinetics, nor convection, nor migration affects the reaction rate. Following the application of a potential step from a value where there is no reaction to one where the concentration of R at the electrode surface is zero, the time dependence of the current will be given by the equation reported by Mahon and Oldham:^23^
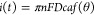
1a
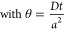
1b
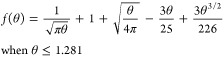
1c
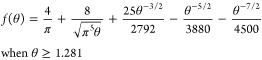
1dwhere *n*, *F*, *D*, *c*, *a*, and *t* are respectively, the number of electrons in the oxidation, the Faraday constant, the diffusion coefficient and bulk concentration of R, the disc radius, and the time when the current is measured. Equation 1a−1d gives the current at all times with a maximum error below 0.02% at intermediate times. A simpler equation proposed by Shoup and Szabo^24^ is less accurate at intermediate times with an error reaching 0.6%. As will be shown below, the calibration-free method relies heavily on currents at intermediate times hence we prefer working with (1).

The time dependence of the current is not obvious from (1) but can be understood by comparing with the diffusion controlled current at a spherical electrode:
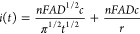
2where *A* is the electrode geometric area and *r* its radius. At short times, the diffusion layer is smaller than the electrode radius, the electrode operates under planar diffusion control, and the current is determined by the first term in (2). This is akin to the classical Cottrell equation for planar electrodes,^25^ and the current drops as *t*^–1/2^. At long times, the diffusion layer is much larger than the electrode radius; this corresponds to spherical diffusion and the current reaches a steady state value given by the second term in (2). At intermediate times, the current reflects the evolution of the diffusion regime from planar to spherical diffusion and is given by both terms in (2). Although (1) is much more complicated because it accounts for the nonuniform distribution of diffusion rates across the disc radius due to edge effects (the flux of reactant reaching the disc is much larger at the edge of the disc than at its center), it reflects a similar evolution of the diffusion regime. From short to intermediate times, the disc current is controlled by (1c) which reflects the switch from planar diffusion to quasi hemispherical diffusion. Then, from intermediate to long times, the current is controlled by (1d) which reflects the switch from quasi hemispherical to hemispherical diffusion. At long times, the disc current reaches a steady state value given by^25^
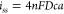
3

**Figure 1:**
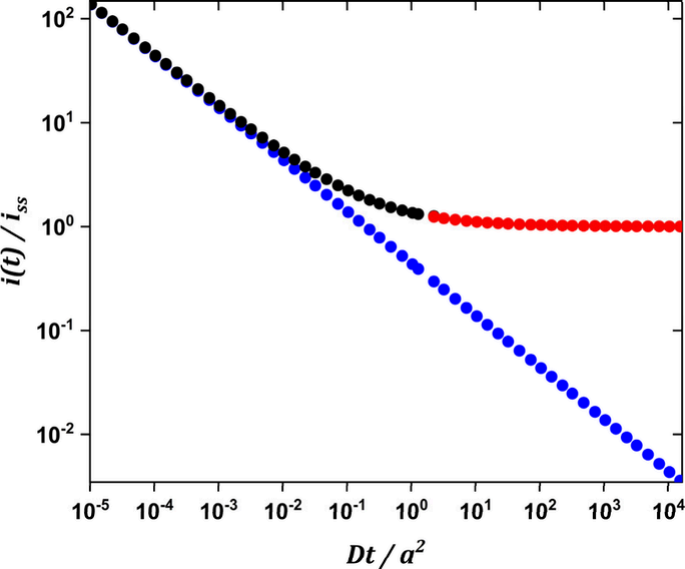
Current values calculated using the Cottrell equation (blue dots), and the Mahon and Oldham equation for short times (black dots) and for long times (red dots). *D* = 5 × 10^–6^ cm^2^ s^–1^ and *a* = 12.5 × 10^–4^ cm.

Evolution of the current through the diffusion regimes is illustrated in Figure 1. At short times (*Dt*/*a*^2^ ≤ 10^–3^), the microdisc current is identical to the Cottrell current thereby reflecting planar diffusion. At intermediate times (10^–3^ ≤ *Dt*/*a*^2^ ≤ 10), the current diverges from the Cottrell current and gradually becomes independent of time. At long times (10 ≤ *Dt*/*a*^2^), the current reaches its steady state value thereby reflecting hemispherical diffusion.

Equation 1a clearly shows that the current is always proportional to the concentration but, to our knowledge, only the steady state current has been exploited for quantitative purposes. For example, we have developed a dissolved oxygen sensor^26^ for oceanographic applications^27^ based on the limiting current for the oxygen reduction on Pt microdisc electrodes, and methods for Cd^2+^, Pb^2+^ and Cu^2+^ quantification in rainwater,^21^ trace nitrite in water and saliva,^28^ and ascorbic acid in acidic extracts of leaves.^29^ Other examples include the determination of the total concentration of redox-active species in liquor,^30^ histamine and glucose with a microelectrode array,^31^ and putrescine.^32^

Knowledge of the diffusion coefficient and microelectrode radius is required to use (3) directly in quantification without the need to prepare a calibration curve. The radius is typically known from the fabrication stage and confirmed by electron microscopy. The diffusion coefficient is rarely known a priori since it depends on the species of interest via its hydrodynamic radius but also on the medium properties such as viscosity, temperature, ionic strength, etc.^33^ Several works present strategies to determine the diffusion coefficient. These strategies usually rely on complex methods and calibrations with substances that have a known *D* value, such as the use of the twin-electrode thin-layer electrochemistry^34^ or an electrochemical system consisting of a pair of electrodes (one macro and one micro).^22^ A simple strategy to obtain the diffusion coefficient was presented by Denuault and co-workers, who proposed the direct determination of *D* through a single microelectrode chronoamperogram.^35^ Their approach relies on the changing dependence of the current with respect to *D* as time increases. At very short times *i* ∝ *D*^1/2^ because of planar diffusion, while at very long times *i* ∝ *D* because of hemispherical diffusion. Normalizing the current with respect to *i*_*ss*_ removes the dependence on *n* and *c* and yields a simple approach to determine *D* from the dependence of the normalized current on *t*^1/2^. The only variable that needs to be known in advance is the disc radius *a*.

If *n* is known, eq 1 can be easily used to derive the concentration if the diffusion coefficient (*D*) is available, which is certainly not the case in several situations. This is especially true when the sample composition or viscosity is very different from those where the *D* value is usually obtained (for instance, aqueous medium with low chemical complexity). Yung and Kwak exploited the Shoup and Szabo equation to determine *D* and *c* but focused their analysis on the determination of *D*. In contrast, our work exploits the more accurate Mahon and Oldham equation and explicitly focuses on the determination of *c*. No one has, to our knowledge, investigated the conditions necessary to operate a reliable calibration-free method.^36^ Furthermore, section SI-4 extends the approach to several redox species and proposes two protocols depending on the redox wave positions. For well separated waves, the approach yields the concentration of each species whereas for overlapping waves, the approach yields the sum of the concentrations.

### The Importance of Time scales

Returning to Figure 1, we can estimate the time scale of each diffusion regime for typical conditions i.e., *D* = 5 × 10^–6^ cm^2^ s^–1^ (typical for a redox species in an aqueous medium), and *a* = 12.5 × 10^–4^ cm (the 25 μm diameter Pt disc is a very common microelectrode). This gives a characteristic diffusion time, *a*^2^/*D*, of ca. 0.3 s. Planar diffusion will therefore dominate at times below 10^–3^*a*^2^/*D*, i.e., below 0.3 ms, and hemispherical diffusion at times above 10 *a*^2^/*D*, i.e., above 3 s. Distortion of the current by double layer charging is not critical here because the geometric area of a microdisc electrode is so small that, even accounting for surface roughness, the double layer charging process is complete in a few μs (the time taken to fully charge the double layer is τ = 5*R*_*S*_*C*_*dl*_, where *R*_*S*_ is the solution resistance and *C*_*dl*_ the double layer capacitance.^25,37,38^ For a typical 25 μm Ø Pt disc with a roughness factor, *R*_*f*_, of 3 and a double layer capacitance, *C*, of 20 μF cm^–2^, immersed in a 0.5 mol L^–1^ NaCl solution, conductivity of *K* = 0.0632 Ω^–1^ cm^–1^, τ = 5*R*_*f*_*πaC*/(4*K*) ≅ 5 μs where *a* is the disc radius).^25,37^ Moreover, as few commercial electrochemical workstations can sample currents below 1 ms, acquiring the current under planar diffusion conditions is generally challenging. It is much easier to acquire the current above 3 s and measure the steady state current before the onset of natural convection, typically a few tens of s. This is easy with small microdiscs as *a*^2^/*D* decreases rapidly with the disc radius. Microdiscs with *a* > 50 μm are too large to reach a diffusion controlled steady state before the onset of natural convection. Most current transients will therefore be acquired at intermediate times, i.e., under quasi hemispherical diffusion, where both terms in (1), (1c) and (1d), will contribute to the current. This is why we recommend using (1) (largest error below 0.02%) rather than the expression from Shoup and Szabo (largest error ca. 0.6%).

### Recommended Protocol

To determine *c* without calibration, one needs to apply a potential step sufficiently large to drive the electrode surface concentration of the species of interest to zero and acquire the current transient from a few ms to a few s, ideally with an acquisition rate between 500 and 1000 points per second to produce a useful data set. The concentration and diffusion coefficient can then be obtained by fitting the current transient to (1) using nonlinear regression. This approach is powerful since it allows fitting several transients at once to account for experimental variations; it is, however, cumbersome and we recommend the simpler procedure shown below.

Since in most cases the current will be acquired at intermediate and long times, i.e., when *t* ≥ 1.281 *a*^2^/*D*, ca. 0.4 s for the conditions shown in Figure 1, eq 1 can be simplified to
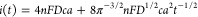
4awhere only the first two terms of (1d) have been retained to provide a linear dependence of the current on *t*^–1/2^. Linear regression of the current transient with respect to *t*^–1/2^ yields the slope and intercept:
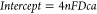
4b
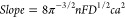
4cand the values of *D* and *c* can easily be derived by solving the system of two equations, only *n* and *a* need to be known.

Although more straightforward than fitting the current transient to the complete form of (1) and extracting *D* and *c* via nonlinear regression, this approach demands reasonable prior knowledge of *D* to ensure that currents are recorded at times such that *t* ≥ 1.281 *a*^2^/*D*. A good approximation of *D* is often available and this is therefore not an issue.

## Experimental Section

### Apparatus and Electrodes

All electrochemical measurements were performed using an Autolab PGSTAT128 bipotentiostat/galvanostat (Metrohm Autolab, Netherlands) equipped with an ultralow current ECD module and the data acquisition software Nova (v.1.11). Current values were measured in the autoranging mode (from 1 μA to 1 nA), with an interval time of ∼2.2 ms for the carbon fiber microdisc (22 ms for the gold microdisc) and high stability bandwidth filtering (HSTAB). The interval time of 22 ms was also used for the quantification of paracetamol using the carbon fiber microdisc. The HSTAB setting is most appropriate for low frequency measurements and suitable for the typical time scales required to fit (4a) to the current transient; this setting significantly reduces noise in the current and potential signals. If shorter time scales are required, e.g., when using very small microdiscs, then the current should be recorded with a higher bandwidth such as the high-speed setting of the Autolab. Acquisition parameters differ between electrochemical workstations; hence we recommend checking the acquisition settings to ensure the current is acquired in the best conditions. Here, for example, we validated the autoranging mode by recording the current transient for the charge of a capacitor in series with a resistor, both selected to cover the relevant time scale of 1 ms to 10 s. With the instrument, software, and settings used, we found that five current values deviate from the data set when the autoranging procedure switches to the higher current sensitivity, and that the current trend recorded at high sensitivity is faithful to that recorded at low sensitivity.

The experiments were performed with two electrodes, a Ag|AgCl (sat. KCl) reference electrode and a microdisc working electrode. The latter, was made with a carbon fiber or gold wire as described previously.^39^ Briefly, a 30 μm diameter carbon fiber was fixed to a NiCr wire with a conductive silver epoxy resin. The bottom part (5 mm) of the fiber was then sealed with an insulating epoxy resin inside a plastic pipet tip. After the resin had set, the pipet was filled with carbon black to secure the electrical connection between the fiber and the NiCr wire. The top of the pipet tip was sealed with parafilm to prevent the carbon black from falling, and to stabilize the NiCr wire. The microdiscs were later polished with increasing grades of sandpaper (320–1200) then alumina powder (0.05 μm) over a polishing cloth. Finally, the microelectrodes were rinsed with water and sonicated for 5 min in distilled water. Scanning electron microscopy was used to assess the surface of the microelectrodes. Figure S1, shows that the geometry of the carbon fiber microelectrode is consistent with a 14.8 ± 0.5 μm radius disc. Additionally, the electrode radius was determined by cyclic voltammetry in a ferricyanide solution with 0.1 mol L^–1^ KCl as a supporting electrolyte, Figure S2. The ferricyanide diffusion coefficient is well established for these conditions: *D* = 7.2 × 10^–6^ cm^2^ s^–1^ at 25 °C.^34^ The voltammogram shows the expected sigmoidal profile and the disc radius, calculated from eq 3 for 5 independent experiments, 14.1 ± 0.1 μm, is in good agreement with the value found by SEM (14.8 ± 0.5 μm, Figure S1). The value obtained by cyclic voltammetry was used in all subsequent calculations, as it represents the electroactive radius of the microdisc, which can be monitored with greater ease and practicality.

The reference electrode was fabricated by electrodepositing AgCl on a silver wire, which was then placed inside a polypropylene pipet with its tip plugged with a permeable polyethylene membrane (mean pore size = 5 μm). The polyethylene membrane was fixed under pressure, by squeezing it through the pipet. The pipet was then filled with a KCl-saturated solution.^40^

The viscosity measurements were performed using a capillary viscosimeter AVS350 TC-Ubbelohde (Schott-Geräte) with 15 min of preconditioning step.

### Reagents and Solutions

All reagents were of analytical grade and used without further purification. Potassium ferricyanide, hexaammineruthenium(III) chloride, ethylene glycol, ascorbic acid, and potassium chloride were obtained from Sigma-Aldrich (St Louis, USA). The food supplement containing ascorbic acid and a pain killer containing paracetamol were purchased from a local drugstore. The solutions were prepared using Nanopure Infinity (Barnstead, USA) purified water.

Potassium ferricyanide (K_3_[Fe(CN)_6_]) and hexaammineruthenium(III) chloride ([Ru(NH_3_)_6_]Cl_3_) solutions were prepared in a 0.1 mol L^–1^ KCl medium. Unless stated otherwise, all solutions were degassed with argon for 15 min to remove dissolved oxygen before carrying out the experiments. To investigate whether the proposed approach would reliably determine the concentration of the target analyte irrespective of its diffusion coefficient, ethylene glycol was added to increase the viscosity of the ascorbic acid solutions (5% (m/v)) and thus decrease the diffusion coefficient of ascorbic acid.

### Analytical Procedures

The ascorbic acid (AA) concentration was determined by chronoamperometry at a potential of 1.1 V. After each experiment, an electrochemical cleaning pretreatment was performed by applying −1.5 V for 10 min to remove any adsorbed species on the electrode surface. Then, a voltammogram was recorded in a potassium ferricyanide +0.1 mol L^–1^ KCl solution to confirm the surface was restored to its initial condition. A voltammogram with a well-defined sigmoidal shape, minimal hysteresis, and the expected limiting current were the conditions used to confirm the microelectrodes were well-behaved electrochemically. If electrochemical cleaning was unsatisfactory, the microelectrode surface was polished again with 0.05 μm alumina and rinsed with distilled water. Although these steps appear cumbersome and time-consuming, the cleaning of the electrode could be automated to include mechanical polishing, a separate cleaning solution, and even an automatic analysis of the shape of the voltammogram to assess the cleanliness of the electrode surface. Alternatively, each current–time data set could be recorded with a single use microelectrode.

Where required, the ascorbic acid concentration was determined by coulometric titration with electrogenerated iodine following the protocol from.^41^ The method consisted of applying a constant current (10.0 mA) in a coulometric cell containing 50 mL of 0.1 mol L^–1^ HAc/Ac- buffer, 2 g of KI, and 1 mL of starch solution. 1.00 mL of the sample or AA solution was also added to the electrochemical cell. At the Pt anode, iodide ions are oxidized to iodine, which chemically reacts with ascorbate near the electrode. The end point is reached when all ascorbate is consumed, and iodine begins to react with starch thereby producing a characteristic blueish color. However, the reaction between AA and starch takes several seconds to produce a noticeable blue coloration. To properly determine the AA concentration, the charge required to generate the blue color in absence of AA was subtracted from the total charge passed to reach the end point. Because electrogenerated iodine can react with hydroxide ions produced at the cathode during water reduction, the Pt cathode was placed in a separate compartment filled with 0.5 mol L^–1^ Na_2_SO_4_ and connected to the rest of the cell via a sintered glass frit. The proposed calibration-free protocol was used to determine the content of ascorbic acid in a food supplement and paracetamol in a medicament. The AA content in an effervescent tablet containing 1.00 g of AA (indicated by the manufacturer on the product label) was quantified after dissolving the tablet in a 100 mL flask for further dilution in the electrochemical cell (1.60 mL sample diluted to 25.0 mL). The analysis of paracetamol was performed by chronoamperometry at a potential of 1.15 V. See Figure S3 for the corresponding voltammogram. The electrochemical cleaning pretreatment was the same as that used in the determination of AA. The paracetamol concentration in a pain killer (whose label concentration was 200 mg/mL) was quantified after dilution in 0.1 mol L^–1^ KCl solution (150 μL sample diluted to 50 mL).

## Results and Discussion

### Proof-of-Concept Experiment

The proposed methodology was first tested using the 1 e^–^ reduction of hexaammineruthenium(III) chloride, eq 5. This well-behaved electrochemical probe undergoes a fast outer-sphere electron transfer and produces diffusion controlled voltammograms, even with experimental conditions yielding high mass transfer coefficients. Its diffusion coefficient is also well-known, *D* = 8.43 × 10^–6^ cm^2^ s^–1^ in 0.1 mol L^–1^ KCl at 25 °C.^42^
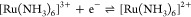
5

**Figure 2:**
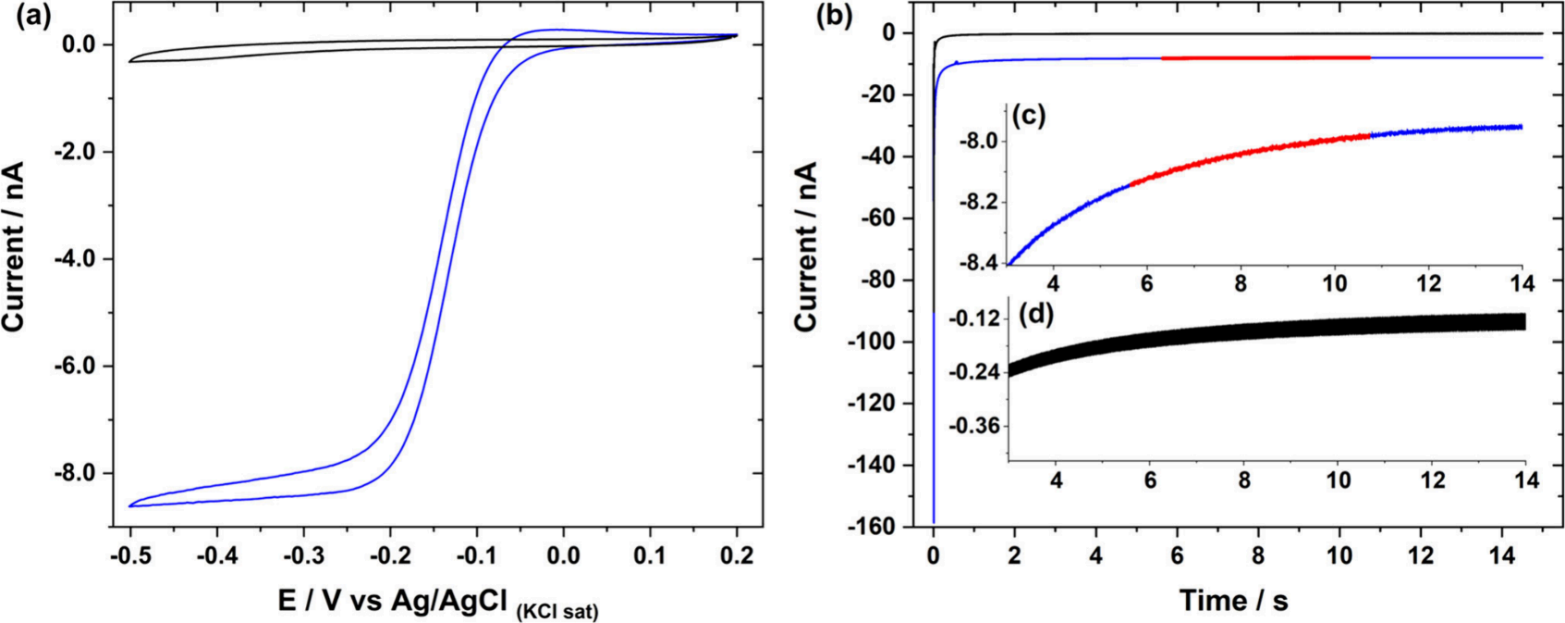
a) Voltammogram recorded with a 14.1 μm radius carbon fiber disc microelectrode in a 1.8 mmol L^–1^ hexaammineruthenium(III) + 0.1 mol L^–1^ KCl solution. Scan rate = 20 mV s^–1^. The black line corresponds to the background CV recorded in the supporting electrolyte. b) Chronoamperogram recorded with the same microelectrode in the same solution when stepping from 0.0 V to −0.4 V. Inset: c) magnification of the current region (red) used to derive *D* and *c*, and d) magnification of the background current.

In Figure 2a, the voltammogram reaches the limiting current from around −0.3 V. Thus, the current transient was acquired by stepping the potential from +0.2 to −0.4 V, Figure 2b. The chronoamperograms were analyzed in the region 5 s ≤ *t* ≤ 11 s, corresponding to 0.4 s^–1/2^ ≤ *t*^–1/2^ ≤ 0.3 s^–1/2^ as shown in Figure S4, i.e., at times long enough that *t* ≥ 1.281 *a*^2^/*D* (0.4 s for the microdisc and redox species used here) as required by eq 4a but short enough not to be affected by natural convection. Although (4a) could have been used from 0.4 s onward, the background current arising from nondiffusion-controlled processes is significant and distorts the chronoamperogram for times lower than 5 s, Figure 2b, therefore we choose to work from this point onward. We believe that this background process is mainly due to the reduction of the carbon functionalities at the electrode surface^43,44^ and that using a more inert electrode material could afford diffusion-controlled currents at shorter times. To assess the influence of the electrode surface, additional experiments with [Ru(NH_3_)_6_]^3+^ were carried out with a gold microelectrode (12.9 ± 0.1 μm radius) starting from 0.0 V and stepping to −0.6 V, Figure S5. In this case, transients could be reliably analyzed over the interval, 3.1 s ≤ *t* ≤ 15.0 s, corresponding to 0.56 s^–1/2^ ≤ *t*^–1/2^ ≤ 0.26 s^–1/2^, Figure S4. Significant extra current was found below 3.1 s, which we believe is due to the reduction of Au oxides formed at the rest potential. This was confirmed by performing experiments (n = 4) starting the potential step from different rest potentials (0.4, 0.2, and 0.0 V). A stable current value was reached at longer times when the experiments were performed using more positive rest potentials. This demonstrates that the electrode material and the state of the electrode surface at rest must be considered to select a time interval over which the current is fully diffusion controlled.

The diffusion coefficient and concentration of [Ru(NH_3_)_6_]^3+^ were calculated for 3 independent experiments, Table S1. The value found for the diffusion coefficient ((7.8 ± 0.2) × 10^–6^ cm^2^ s^–1^) is in good agreement (deviation = −7.5%) with that from the literature, Table S1. Similarly, the value for the concentration of electroactive species (1.78 mmol L^–1^) is close to that expected after dissolving the [Ru(NH_3_)_6_]^3+^ salt in the KCl solution (1.8 mmol L^–1^). Similar experiments were repeated with the gold microelectrode, and the results of 5 independent chronoamperograms yielded values for the concentration and diffusion coefficient of 1.79 ± 0.05 mmol L^–1^ and (8.8 ± 0.3)x10^–6^ cm^2^ s^–1^, respectively, which agree with those found using the carbon fiber microdisc.

### Ascorbic Acid Determination

**Figure 3:**
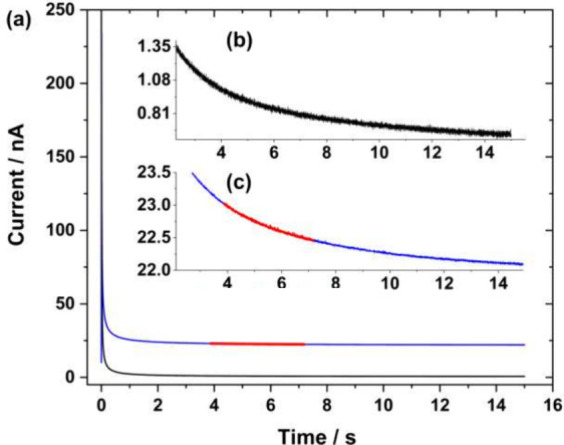
a) Chronoamperometry performed in a 3 mmol L^–1^ ascorbic acid +0.1 mol L^–1^ KCl solution when stepping the electrode from 0.0 V to +1.1 V. The black line corresponds to the background current recorded in the supporting electrolyte. Inset: b) magnification of the background current, and c) magnification of the current region (red) used to derive D and c.


The electrochemical oxidation of ascorbic acid to dehydroascorbic acid occurs by transferring two electrons and losing two protons, eq 6. Although the ascorbic acid oxidation starts at around 0.2 V, the current does not reach a steady-state value (Figure S6) before the water oxidation reaction; therefore, the chronoamperometric analysis was carried out at a potential of 1.1 V, Figure 3a.

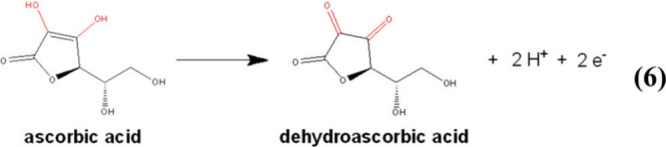
6

**Figure 4:**
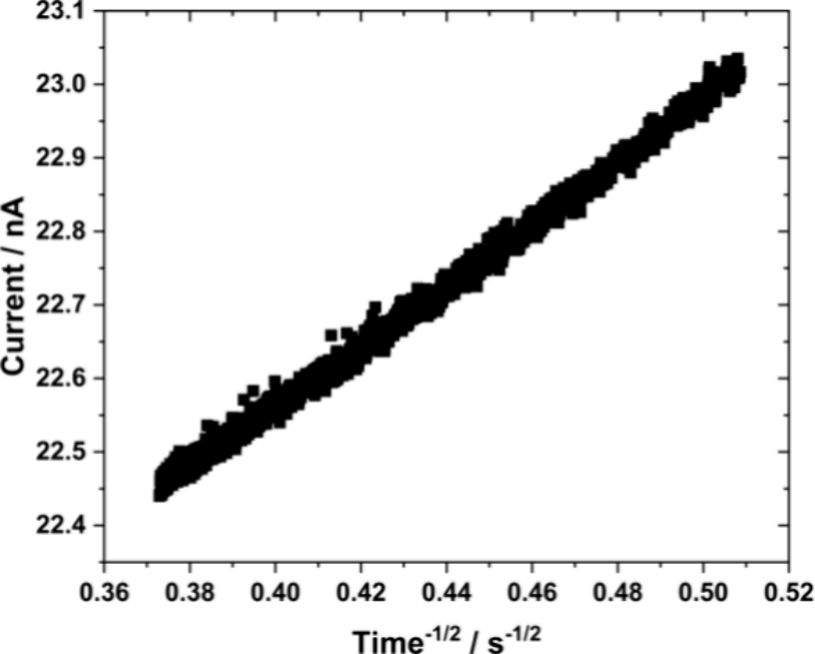
Linearization of the current region highlighted on the chronoamperogram shown in Figure 3. (Current/nA) = 4.14 (time/s)^−1/2^ + 20.90; R^2^ = 0.9951.

The selection of the time interval to perform the linearization is crucial to ensure that eq 4a applies. Here, the chronoamperogram was linearized between ca. 4 and 8 s as highlighted in Figure 3a. This corresponds to 0.51 s^–1/2^ ≤ *t*^–1/2^ ≤ 0.37 s^–1/2^ as shown in Figure 4. The plot shows a very good linear dependence of the current on *t*^–1/2^ thereby confirming the validity of the approach. The concentration of AA was known so the data was analyzed to derive *D* and *n*. The diffusion coefficient found for ascorbic acid in 0.1 mol L^–1^ KCl was ca. 6.6 × 10^–6^ cm^2^ s^–1^, which agrees with values reported in phosphate buffer at pH 7 (5.7 × 10^–6^ cm^2^ s^–1^)^45,46^ and at pH 3.5 (5.77 × 10^–6^ cm^2^ s^–1^).^47^ Similarly, the number of electrons, 1.94, was found to be in close agreement with the expected 2-electron process.

Further experiments (*n* = 5) were performed in ascorbic acid solutions (3 and 4 mmol L^–1^) using the calibration-free method to assess the repeatability (same operator using a single microelectrode), and the results were found to be 3.10 ± 0.09 mmol L^–1^ and 4.2 ± 0.3 mmol L^–1^, respectively (Table 1). Reproducibility tests (measurements made with different microelectrodes or coulometric experiments performed on different days) yielded 2.9 ± 0.3 mmol L^–1^ and 4.2 ± 0.4 mmol L^–1^, respectively. The results obtained with the calibration-free method agree with those obtained by coulometry, Table 1, and present a deviation ≤8.5%.

**Table 1:** Ascorbic Acid Concentrations in Aqueous Medium Found by Coulometry and the Calibration-Free Method, eq 4a (*n* = 5)

	**Repeatability[Reproducibility]**				
**Ascorbic Acid Sample/mmol L**^**–1**^	**Coulometry/mmol L**^**–1**^	**RSD /%**	**Calibration-free/mmol L**^**–1**^	**RSD/%**	**Deviation/%**
3.0	3.02 ± 0.09	3.0	3.10 ± 0.09	2.9	2.6
	[3.17 ± 0.04]	**[1.3]**	[2.9 ± 0.3]	**[10.3]**	**[-8.5]**
4.0	4.0 ± 0.1	2.5	4.2 ± 0.3	7.1	5.0
	[4.3 ± 0.1]	**[2.3]**	[4.2 ± 0.4]	**[9.5]**	**[-2.3]**

The calibration-free approach was further tested by determining the concentration of ascorbic acid in a solution with different viscosity. This was achieved by adding a nonelectroactive thickener (ethylene glycol 5% (m/v)) to the AA solution. Viscosity was selected because it affects the diffusion coefficient and thus could have an impact on the concentration determined by the proposed method. The results from the calibration-free method, Table S2, agree with those obtained by coulometry and give an RSD ≤ 8.8%, demonstrating the usefulness of the proposed approach, even for samples with different viscosities. Linearization of the current transients recorded with the 3 mmol L^–1^ + 0.1 mol L^–1^ KCl solution produced *D* values of (6.2 ± 0.2)x10^–6^ cm^2^ s^–1^ in the absence of thickener and (5.6 ± 0.2)x10^–6^ cm^2^ s^–1^ in the presence of 5% ethylene glycol (m/v). This 9.7% drop in *D* is consistent with the viscosity increase (12.8% (n = 3), from 0.75 ± 0.01 cP to 0.86 ± 0.01 cP) as expected from the Stokes–Einstein equation.^48^ Here, too, the AA concentration determined with the calibration-free method was found to agree with that determined by coulometry, Table S2, thus confirming that the method was immune to a change in viscosity.

Finally, a food supplement and a medication containing ascorbic acid and paracetamol, respectively, were analyzed using the proposed method. Table 2 shows results in good agreement with the values stated on the labels (deviation ≤10.8%).

**Table 2:** Ascorbic Acid and Paracetamol Concentrations in a Food Supplement and a Medication, Respectively, Found by the Calibration-Free Method, eq 4a (*n* = 3)^a^

**Sample**	**Concentration on the label**	**Calibration-free**	**RSD/%**	**Deviation/%**
Food Supplement	0.25 g/tablet	0.277 ± 0.007 g/tablet	2.5	10.8
Medication	200 mg/mL	206 ± 2 mg/mL	1.0	3.0

a The deviation was determined with respect to the value reported by the manufacturer.

## GENERAL CONSIDERATIONS

The results reported above have clearly demonstrated that the proposed method is suitable for measuring concentrations in the mmol L^–1^ range without interference from background electrochemical processes or instrumental considerations, provided the chronoamperograms are analyzed at sufficiently long times for eq 4a to be valid and at sufficiently short times to be unaffected by natural convection. The method is inherently capable of determining lower concentrations; however, the detection limit will depend on the magnitude of the background currents. The total current represents the sum of the background and analyte currents whereas eqs (1 or 4a) only describe the current due to the analyte reaction. Hence, improving the detection limit will require either subtraction of background currents recorded in absence of the analyte of interest or fitting with an equation which accounts for both background and analyte currents. The former is possible but not attractive since the calibration-free method aims to reduce the complexity of the analytical protocol; background subtraction would, for example, preclude continuous monitoring applications. The latter is more attractive because background processes tend to be capacitive in nature and this can be easily accounted for by a simple exponential decay of the current with time (the Faradaic current for reactions involving adsorbed species follows an exponential decay^49^). A new equation can thus be constructed as shown below:
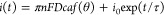
7where *f*(θ) is given by eq 1d (or 1c+1d if applying the complete Mahon and Oldham equation), *i*_0_ is the initial background current, and τ is the time constant for the decay of the background current. With two additional unknowns, *i*_0_ and τ, the calibration-free approach must now rely on nonlinear regression of the chronoamperogram to (7). This can be automated and thus does not preclude continuous monitoring applications. Selection of the time scale for analysis of the current can help minimize the contribution from background processes. Most background processes will be due to the electrochemical reduction or oxidation of species adsorbed or bound to the electrode surface. Typical examples include the formation/stripping of oxides on metallic microelectrodes or the reduction/oxidation of functionalities on carbon fiber microelectrodes. The magnitude of these background currents is proportional to the electroactive area which is significantly decreased with microdisc electrodes. As for double layer charging, the time taken to complete these surface electrochemical reactions is short. Therefore, analyzing the chronoamperogram at long times also ensures that the contribution from the background currents is minimized. It is not possible to give general guidelines for the smallest time from which the current should be analyzed since this will depend on the electrode material, on the electrode electroactive area (hence roughness), on the kinetics of the redox process, and on the potential applied to record the chronoamperograms. This is particularly important for slow electron transfer processes requiring high overpotentials. For instance, we have noticed that the oxidation of ascorbic acid at the carbon fiber microelectrode required a large overpotential, and the limiting current was only reached at a potential where the background current, also likely due to the carbon functionalities, is not negligible. This could be circumvented with a different electrode material or by functionalizing the electrode surface to improve the kinetics of the analyte redox process.^50^ Some attention should also be given to instrumental parameters, the presence of soluble interfering compounds, and electrode surface cleaning, as discussed in section 5 of the SI.

## RECOMMENDATIONS

Four conditions determine the success of the method: 1) the current transients must be recorded accurately; 2) the current must be purely diffusion-controlled; 3) the current transients must be analyzed with an appropriate analytical expression; 4) the electrode surface must be clean and reproducible. To achieve condition 1, users must validate the acquisition parameters, control software, and instrumentation; this is easily done with a dummy cell, e.g., a R-C series circuit with known resistance and capacitance, to produce known current transients. Achieving condition 2 is more challenging as it depends on many parameters such as the target analyte, potential window of interest, electrode material, sample matrix, and sample viscosity. To ensure the current is purely diffusion-controlled, there must be no contribution from background processes and no contribution from convection or migration. To prevent interference from convection, the sample solution must be quiescent, and the measurement must be complete before the onset of natural convection. To prevent migration, the sample should have a large background electrolyte concentration. As we showed above, the contribution from background electrochemical processes can be long lasting. Here, we obtained purely diffusion-controlled current after waiting for a few s, 5 s for [Ru(NH3)_6_]^3+^ and 4 s for ascorbic acid. This will depend on the electrode material (as noted with the gold microelectrode), on the state of the electrode surface, and on the presence of interfering compounds in the sample. Meeting condition 3 is less of a problem but the data analysis must be performed over the correct time scale to match the validity of the equation selected to fit the current transient. If the transient is known to be purely diffusion-controlled, then (1) or (4a) can be selected. If a background electrochemical reaction contributes to the current and there is no possibility of background subtraction, then (7) should be selected. If the target potential must be applied in the rising part of the analyte voltammetric wave, then equation SI-A1 should be selected. Condition 4 can be met in two ways, either via the classical mechanical polishing approach or via an electrochemical conditioning protocol to reliably oxidize or reduce the electrode surface before applying the target potential. While the former is more suited to lab measurements, the latter is suitable for automation and continuous monitoring applications. Once careful work has been carried out upfront to ensure the four necessary conditions are met, this calibration-free procedure can be exploited reliably.

## Conclusions

The calibration-free method described in this work allows fast (under 15 s), reliable, reproducible, low-cost determination of analyte concentrations without using standards. Both the concentration and the diffusion coefficient are obtained through a single chronoamperometric experiment. With far fewer steps than conventional protocols, the method is simple to implement, easily miniaturized on a sensor platform, and suitable for continuous monitoring applications. Being performed with a microelectrode, the method is well suited to in situ measurements in microenvironments, e.g., for the monitoring of species in living organisms.

We also wish to stress a significant difference with the method reported by Denuault et al.^35^ Their protocol involved normalizing the current transient with the steady state current (*i*_*ss*_) to remove the dependence on *n* and *c*. This implies that *i*_*ss*_ can be measured. However, there are situations where this is not possible, e.g. in viscous media. As viscosity increases, the diffusion regime at the microdisc evolves very slowly and it takes a very long time to reach the hemispherical diffusion regime where *i*_*ss*_ can be measured. That can be remediated to some extent by using very small disc radii, but in very viscous media, e.g. with ionic liquids, it is no longer practically possible to establish the hemispherical diffusion regime and *i*_*ss*_ cannot be recorded. Our approach does not require normalization by *i*_*ss*_ and works even in conditions where the hemispherical diffusion regime is not established. Overall, our method goes far beyond the original work of Denuault et al.^35^ Their approach was seminal but did not consider the determination of *c*. While Jung and Kwak reported the determination of *D* and *c* from a single microdisc transient, they did not validate their approach with respect to *c*.^36^ In contrast, our method explicitly focuses on *c*, and proposes conditions where the concentration can be reliably determined from a single transient. To our knowledge, this had not been reported previously.

## Supporting Information Available

Scanning electron microscopy of the surface of the carbon microelectrode, ferricyanide voltammograms, table with results for hexaammineruthenium(III), lineariarization of the chronoamperogram, chronoamperogram recorded with a gold microdisc electrode, ascorbic acid voltammograms, table with results for ascorbic acid concentrations containing a thickener, a discussion about sources of errors, and an extension of the proposed method to cases involving more than one redox species (PDF)
