# Supplementary material for: Calibration-Free Analysis with Chronoamperometry at Microelectrodes
Source: Anal Chem. 2024 Sep 3;96(37):14766–74. doi: 10.1021/acs.analchem.4c01645 (PMC11411494; doi:10.1021/acs.analchem.4c01645)
Supplement: Supplementary file 1 — ac4c01645_si_001.pdf [file ac4c01645_si_001.pdf]

Supporting Information

Calibration-Free Analysis with Chronoamperometry at Microelectrodes

Valdomiro S. Conceição‡, Douglas P. M. Saraiva‡, Guy Denuault#, Mauro Bertotti‡*

‡ Department of Fundamental Chemistry, Institute of Chemistry, University of São Paulo-USP, São Paulo, 05508-000, Brazil

School of Chemistry, University of Southampton, Highfield, Southampton SO17 1BJ, UK

* Email: mbertott@iq.usp.br

Summary

1. Characterization of the Electrode Geometry S-2

Figure S1 - Scanning Electron Microscopy (SEM) of a disc microelectrode. S-2

Figure S2 – Voltammograms recorded with a microelectrode. S-2

2. Analytical Procedures S-3

Figure S3 – Voltammogram in a 3 mmol L^{-1} paracetamol solution S-3

Equation S1 – Electrochemical oxidation of paracetamol S-3

3. Proof-of-Concept Experiment S-4

Table S1 – Diffusion coefficient and concentration found for hexaammineruthenium (III) by the calibration-free method. S-4

Figure S4 – Linearization of the transient region of the chronoamperogram. S-4

Figure S5 – Chronoamperogram recorded with a gold microdisc electrode. S-5

4. Ascorbic Acid Determination S-5

Figure S6 – Voltammogram recorded with a carbon fibre microdisc. S-5

Table S2 – Ascorbic acid concentrations in solutions containing a thickener found by coulometry and the calibration-free method. S-6

5. General Considerations S-6

6. References S-10

1. Characterization of the Electrode Geometry

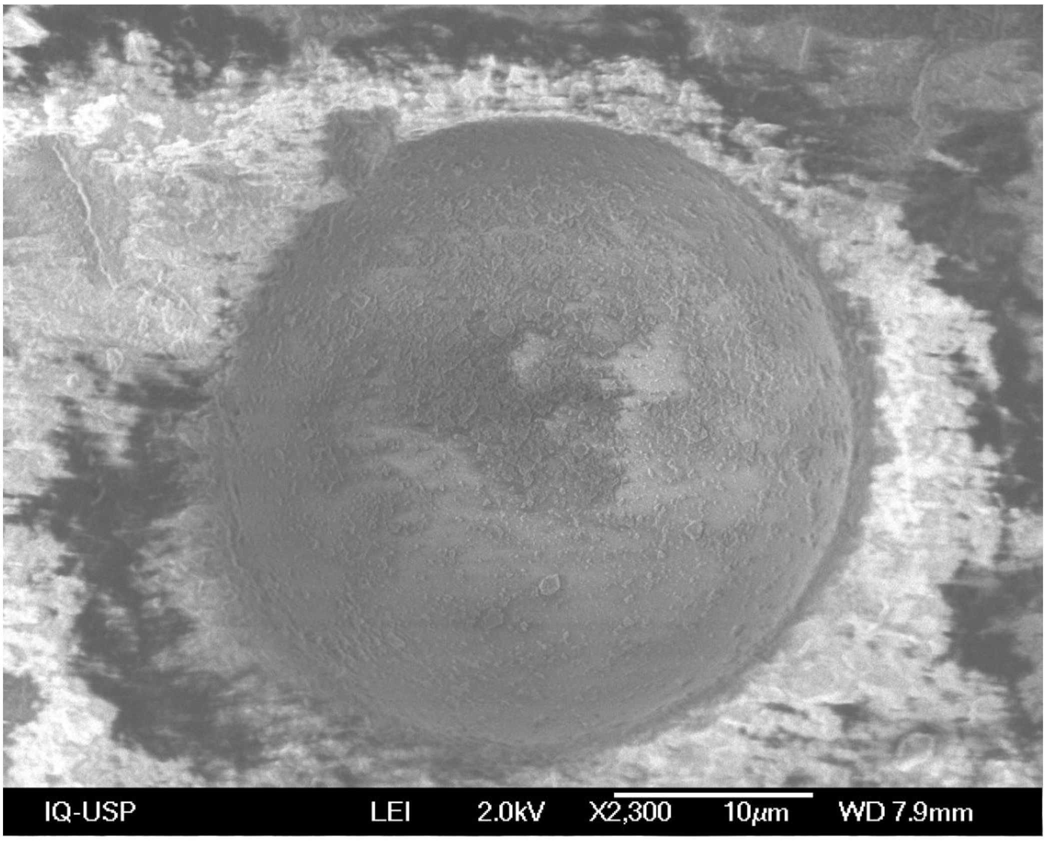

Figure S1 *- Scanning Electron Microscopy (SEM) of a carbon fibre disc microelectrode.*

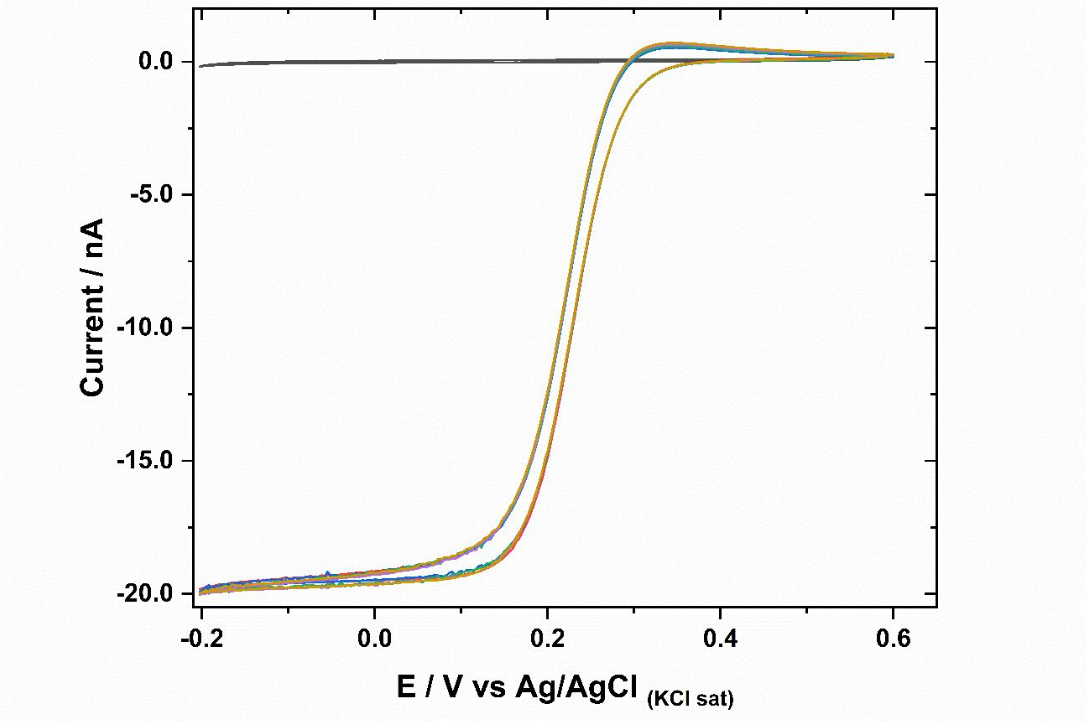

Figure S2 *– Five voltammograms recorded with a 14.1 μm radius carbon fibre disc microelectrode in 5.00 mmol L^{-1} ferricyanide + 0.1 mol L^{-1} KCl. Scan rate = 20 mV s^{-1}. The dark grey line corresponds to the background CVs recorded in the supporting electrolyte.*

2. Analytical Procedures

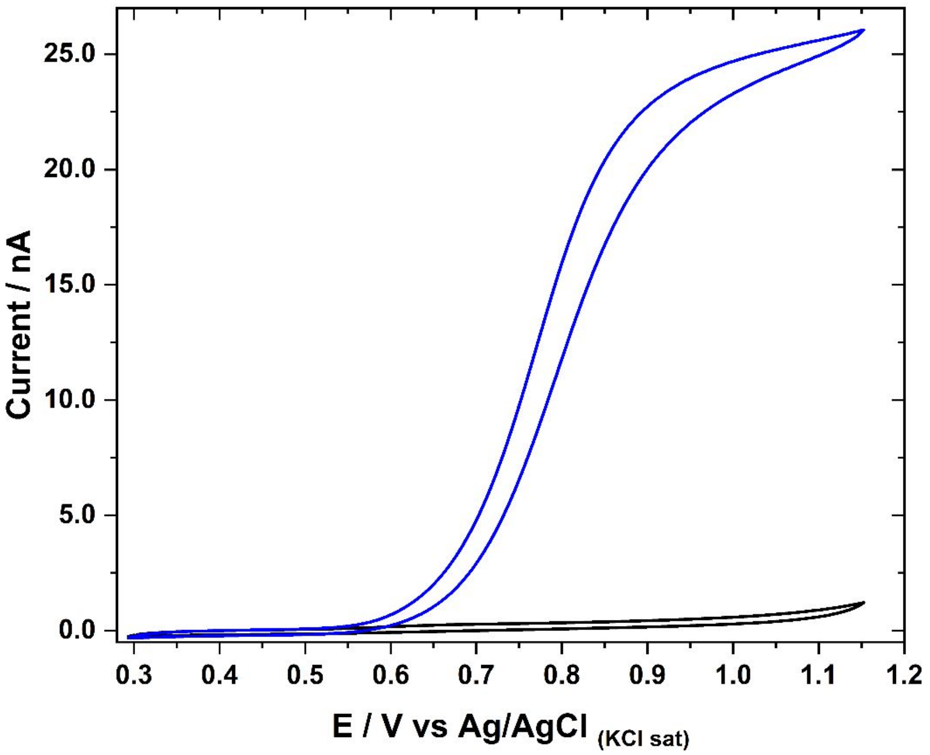

Figure S3 *– Voltammogram recorded with the carbon fibre microdisc (14.1±0.1 µm) in a 3 mmol L^{-1} paracetamol + 0.1 mol L^{-1} KCl solution. Scan rate = 25 mV s^{-1}. The black line corresponds to the background CV recorded in the supporting electrolyte*

Equation S1 *– Electrochemical oxidation of paracetamol to N-acetyl-p-benzoquinone imine (NAPQI)*

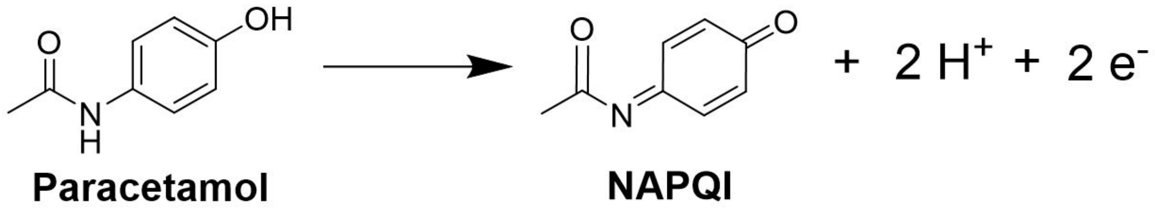

3. Proof-of-Concept Experiment

Table S1 – *Diffusion coefficient and concentration found with equation 4a from chronoamperometric experiments (n=3) performed in a 1.8 mmol L^{-1} hexaammineruthenium(III) + 0.1 mol L^{-1} KCl solution with a 14.1 μm radius carbon fibre disc. * from ref. [1].*

Variable	Expected value	Experimental value	RSD (%)	Deviation (%)
10^6 D / (cm^2 s^{-1})	8.43*	7.8±0.2	2.6	-7.5
c / (mmol L^{-1})	1.8	1.78±0.03	1.7	-1.1

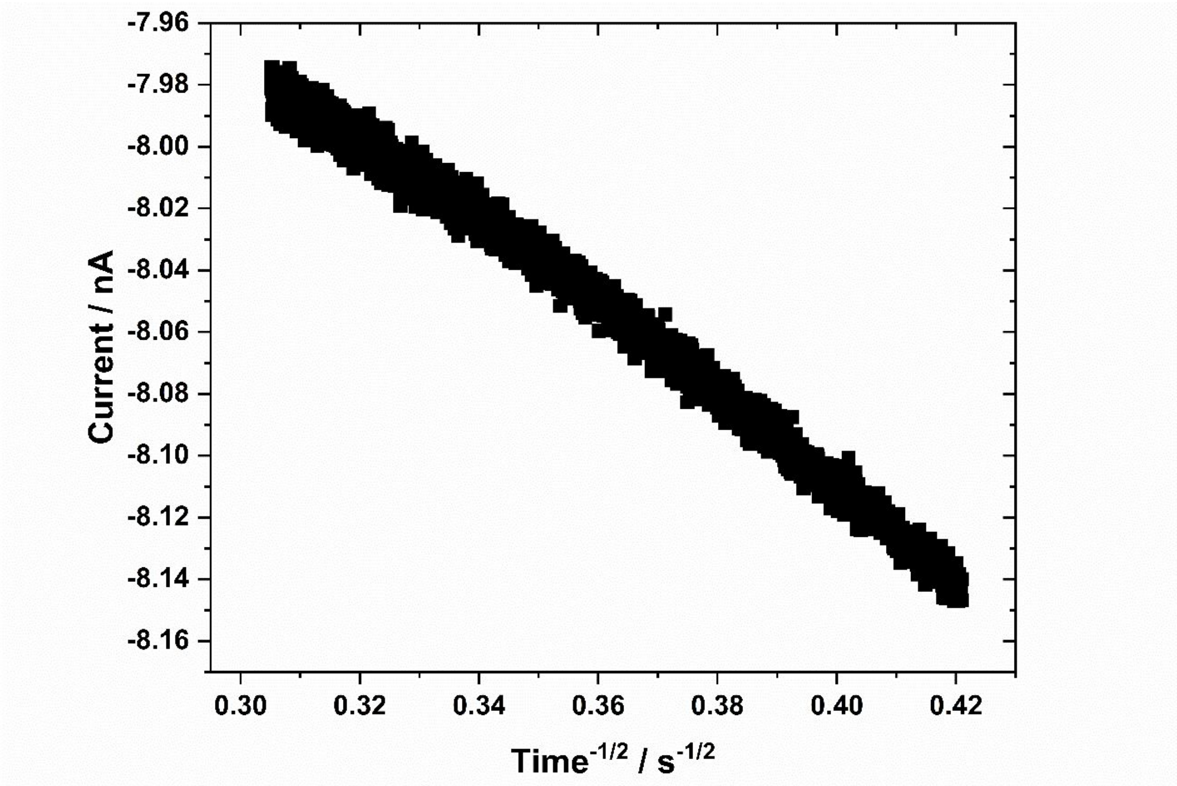

Figure S4 – *Linearization of the transient region highlighted on the chronoamperogram of Fig. 2b. (Current / nA) = -1.39 (time / s)$^{-1/2}$ - 7.55; R^2 = 0.9895.*

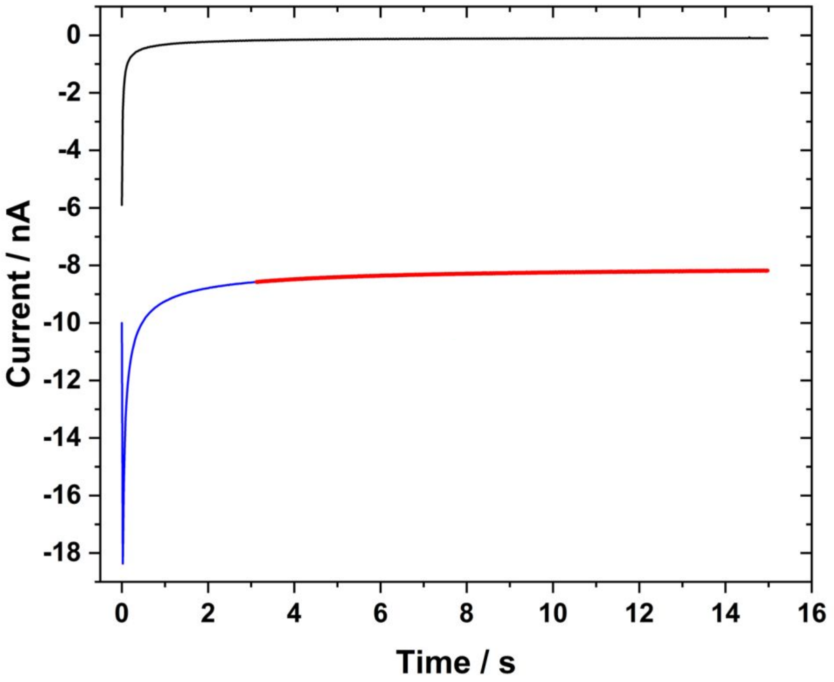

Figure S5 – *Chronoamperogram recorded with a gold microdisc electrode, 12.9±0.1 μm radius, in a 1.8 mmol L^{-1} hexaammineruthenium(III) + 0.1 mol L^{-1} KCl solution when stepping from 0.0 V to -0.6 V. The black line corresponds to the background current recorded in the supporting electrolyte. The region selected to analyse the current is highlighted in red.*

4. Ascorbic Acid Determination

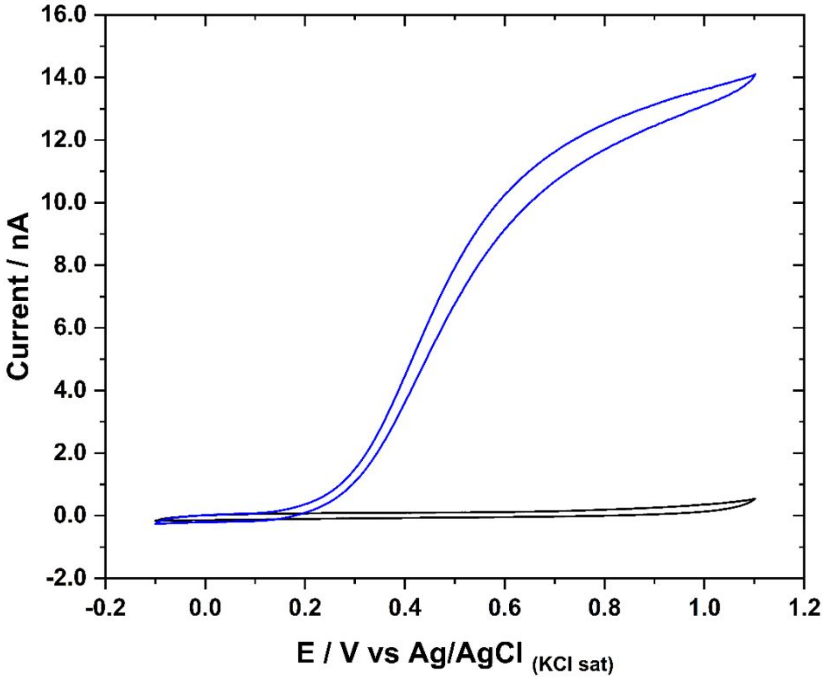

Figure S6 – *Voltammogram recorded with the carbon fibre microdisc (14.1±0.1 μm) in a 2 mmol L^{-1} ascorbic acid + 0.1 mol L^{-1} KCl solution. Scan rate = 25 mV s^{-1}. The black line corresponds to the background CV recorded in the supporting electrolyte.*

Table S2 – *Ascorbic acid concentrations in solutions containing a thickener (ethylene glycol 5% (m/v)) found by coulometry and the calibration-free method, equation 4a (n=5). The deviation corresponds to measurements obtained with the two methods.*

**Ascorbic Acid Sample / mmol L^{-1}**	Repeatability **[Reproducibility]**				
	**Coulometry / mmol L^{-1}**	**RSD / %**	**Calibration-free / mmol L^{-1}**	**RSD / %**	**Deviation / %**
3.0	3.07±0.07 **[3.17±0.08]**	2.3 **[2.5]**	2.8±0.1 **[3.4±0.3]**	3.6 **[8.8]**	-8.8 **[-2.2]**
4.0	4.2±0.1 **[4.2±0.1]**	2.4 **[2.4]**	4.1±0.2 **[4.4±0.2]**	4.9 **[4.5]**	-2.4 **[4.8]**

5. General Considerations

Some attention should also be given to instrumental parameters, as the potentiostat must be capable of measuring currents with good precision over a wide range of values and with an accurate time base. Using dummy cells, we have evaluated that our potentiostat accurately acquires data with good precision in most acquisition modes. Although fast data acquisition and no filtering are advised, current auto-ranging, which usually applies to low acquisition rates, leads to better chronoamperometric results when the experiment generates a wide range of current values. With our instrument and settings used, auto-ranging lead to a loss of about five data values when the switching between ranges took place, but this had no effect on the time base and the current trend recorded after switching to the higher sensitivity was faithful to that recorded before switching. Auto-ranging is particularly important when fitting with the whole Mahon and Oldham equation since the current decreases by several orders of magnitude before reaching its limiting value. For typical conditions, Figure 2b and 3a, the current decreases by over one order of magnitude between 1 ms and 10 s. A logarithmic operational amplifier-based current follower would address this type of instrumental issue but, to our knowledge, this is not available on commercial electrochemical workstations.

The presence of soluble interfering compounds, oxidized or reduced concomitantly with the analyte, is also of great concern, mainly when high overpotentials must be applied to reach the limiting current, such as for ascorbic acid. This will strongly depend on the difference between the potential required to reach the limiting current for the analyte and the position of the voltammetric wave

for the interfering species. One way to address this issue is to apply a target potential in the rising part of the analyte voltammetric wave, e.g., at the half-wave potential, and correct for this in the expression used to fit the faradaic response of the analyte as shown below:

$$i(t) = \pi\, n\, F\, D\, c\, a\, f(\theta)\left(\left(1 + exp\left(-\frac{nF}{RT}(E - E^{0\prime})\right)\right)^{-1}\right) \qquad \text{(A1)}$$

where R and T have their usual meaning, E is the potential applied to record the chronoamperogram, and $E^{0\prime}$ is the formal potential for the oxidation / reduction of the analyte. The correcting factor simply accounts for the fact that the surface concentration of the analyte is not zero at the applied potential and it is derived from the Nernst equation assuming that the oxidised and reduced forms of the analyte share the same diffusion coefficient. This approach could easily decrease the overpotential needed for the analyte by 100 to 200 mV and significantly decrease the extra current; however, it introduces an additional unknown, $E^{0\prime}$, which would need to be estimated from voltammetry.

Although we have advocated the use of equation 4a because it yields a simple linear relationship between the current and the inverse square root of time, the concentration and diffusion coefficient values can also be obtained by non-linear regression of the experimental current transient to equation 1, having set the values for n and a as constants and those of D and c as adjustable variables. This can be performed easily on commercial software such as Origin, but it is helpful to start with estimates of the concentration and diffusion coefficient to guide the regression away from non-sensical values. The benefit of this approach is that it allows fitting several transients at once to the same equation which is advantageous to account for experimental variability. Furthermore, the non-linear regression routines can account for experimental errors on the current from a dataset or from a mathematical expression. Although the calibration-free approach based on equation 4a has obvious simplicity, the advantages of the non-linear regression of the chronoamperograms to equation 1 should not be neglected.

As with all amperometric techniques, the approach proposed here works provided the electrode surface is clean and reproducible. For lab measurements, this is easily achieved by polishing the electrode surface as reported above and it is advisable to acquire voltammograms to assess the state of the electrode surface. However, manual cleaning is not possible with embedded sensor

platforms used for continuous monitoring. For these applications, the electrode surface can be reconditioned electrochemically using a suitable potential waveform. In the study presented here, we simply held the electrode potential at a very negative value for a few minutes before applying the target potential. In a previous work, we implemented a potential waveform to recondition the electrode surface and monitor the dissolved oxygen concentration in the ocean [2,3]. In this case, the electrode was briefly held at a slightly oxidising potential to form a small coverage of oxide and strip any adsorbed species, then the target potential was applied to reduce oxygen and acquire the current transient. Once the conditioning waveform has been optimised for the sample conditions, it can be integrated within the analytical sequence to form an automated measurement protocol.

It is interesting to consider situations where several redox species are present in the solution. Let us assume that two reduced species R_1 and R_2 are in the solution. We respectively define their number of electrons, concentration, diffusion coefficient, and oxidation halfwave potential as n_1, c_1, D_1, $E_1^{1/2}$, n_2, c_2, D_2 and $E_2^{1/2}$. Two limiting cases arise depending on $E_1^{1/2}$ and $E_2^{1/2}$.

If the oxidation waves are far apart, say $E_1^{1/2} = E_2^{1/2} - 400$ mV, it is possible to select a target potential where only R_1 is consumed at the electrode. This generates a chronoamperogram, $i_1(t)$, only determined by n_1, c_1, and D_1. At this point, c_1 and D_1 can be determined with the method proposed in the main part of the article. Applying a 2nd potential step from a value where no reaction occurs to one where R_1 and R_2 are both consumed at the electrode, produces a chronoamperogram, $i_{tot}(t)$, determined by n_1, c_1, D_1, n_2, c_2 and D_2. Since fluxes are additive, subtracting $i_1(t)$ from $i_{tot}(t)$ yields a new transient solely related to n_2, c_2 and D_2 (this can be easily validated by simulations). The new transient can then be analysed with the proposed method to determine c_2 and D_2. Here the values of n_1 and n_2 do not influence the outcome of the analysis since the first chronoamperogram is analysed separately from the second. While the approach could be extended to more than two species, in practice this would only work if the respective oxidation waves were sufficiently far apart for the chronoamperograms to be associated with one extra species at a time.

However, if the oxidation waves are very close to each other, say $E_1^{1/2} = E_2^{1/2}$ -50 mV, it is no longer possible to select a target potential to consume only R_1 and derive c_1 and D_1. Whatever the target potential, there is always a significant flux of R_1 and R_2. Stepping from a potential where no reaction occurs to one where R_1 and R_2 are both fully consumed at the electrode produces a chronoamperogram reflecting an apparent concentration, c_{ap}, and apparent diffusion coefficient, D_{ap}, both linked to c_1, D_1, c_2 and D_2. Since the microdisc diffusion regime evolves over time, it is difficult to extract a clear relationship between the apparent D and c values and those of the respective species. However, at long times, the diffusion regime is virtually hemispherical, the hemispherical diffusion layer is close to its asymptotic value, and the total current approaches the sum of two limiting currents

$$i_{tot} = i_{lim,1} + i_{lim,2} = 4nFD_1c_1a + 4nFD_2c_2a \quad \text{(A2)}$$

Here we have assumed that $n_1 = n_2 = n$ for the sake of simplicity. We also have

$$i_{tot} = 4nFD_{ap}c_{ap}a \quad \text{(A3)}$$

Hence

$$D_1c_1 + D_2c_2 = D_{ap}c_{ap} \quad \text{(A4)}$$

Since the fluxes are additive and the diffusion layer thickness is similar for both species we have

$$c_1 + c_2 = c_{ap} \quad \text{(A5)}$$

and therefore

$$\frac{D_1c_1+D_2c_2}{c_1+c_2} = D_{ap} \quad \text{(A6)}$$

Both D_{ap} and c_{ap} are easily derived by fitting the overall chronoamperogram to equation 4a of the main article. The above procedure can also be extended to more than two species but, in this case, it only works if the n values are identical.

In summary, when the two oxidation waves are far apart, the proposed method can be used to derive the concentration and diffusion coefficients of both redox species using two chronoamperograms, one with a target potential sufficient to fully consume R_1 and the other with a target potential set to fully consume R_1 and

R_2. In contrast, when the oxidation waves are very close, typically less than 400 mV apart, the proposed method can be used to derive the sum of the concentrations of the redox species from a single chronoamperogram recorded with a target potential sufficiently large to fully consume both species. Although not shown here, we have checked the protocol for both cases using simulations of the chronoamperograms for the oxidation of two redox species on a microdisc electrode.

6. References

(1) Wang, Y.; Limon-Petersen, J. G.; Compton, R. G. Measurement of the Diffusion Coefficients of [Ru(NH3)6]3+ and [Ru(NH3)6]2+ in Aqueous Solution Using Microelectrode Double Potential Step Chronoamperometry. J Electroanal Chem 2011, 652 (1–2), 13–17.

(2) Sosna, M.; Denuault, G.; Pascal, R. W.; Prien, R. D.; Mowlem, M. Development of a Reliable Microelectrode Dissolved Oxygen Sensor. Sens Actuators B Chem 2007, 123 (1), 344–351.

(3) Sosna, M.; Denuault, G.; Pascal, R. W.; Prien, R. D.; Mowlem, M. Field Assessment of a New Membrane-free Microelectrode Dissolved Oxygen Sensor for Water Column Profiling. Limnol Oceanogr Methods 2008, 6 (4), 180–189.
